# An overview and trend analysis of research on the relationship between urban streets and residents' health in China pre- and post COVID-19 pandemic

**DOI:** 10.3389/fpubh.2023.1126656

**Published:** 2023-03-23

**Authors:** Lingchao Meng, Kuo-Hsun Wen

**Affiliations:** ^1^Faculty of Humanities and Arts, Macau University of Science and Technology, Taipa, Macau SAR, China; ^2^School of Design, Fujian University of Technology, Fuzhou, China

**Keywords:** COVID-19, urban streets, residents' health, overview and trend analysis, bibliometric analysis

## Abstract

**Introduction:**

After the outbreak of COVID-19, the international community has been faced with various problems it has brought to cities. A large number of research projects and corresponding management measures were launched globally, trying to reduce the impact of COVID-19 on society. Among them, exploring how to maintain the health of residents by managing and updating the design of urban streets is one of the important issues regarding urban sustainability in the post-epidemic era.

**Methods:**

This study uses bibliometric analysis techniques to obtain an overview of the knowledge structure of 898 Chinese urban streets and residents' health relationship studies from the China National Knowledge Infrastructure (CNKI) database for two periods (1999–2019 and 2020–2022). Five aspects were analyzed in terms of the keyword domain co-occurrence network, topic evolution path, emergent terms, hierarchical clustering, and confusion matrix.

**Results and discussion:**

The findings revealed that studies focused on six broad themes: community residents, health surveys, health education, COVID-19, healthy city, and public health. Based on these findings, the paper compares and discusses research priorities before and after the outbreak and highlights areas for further research and attention.

## 1. Introduction

Historically, cities have been at the center of pandemics, and public health crises have played a significant role in the evolution of urban planning and design concepts and technologies (1). As continuously suffering for almost 3 years, COVID-19 can possibly cause more than 100 million people to go into extreme poverty worldwide, which can be the “worst setback in decades” (2). The pandemic forces people to re-examine society as it exacerbates inequality and demonstrates the inadequacy of urban governance (3). China, with a total population of 18.47% of the world's total population, has 60.8% of its population living in cities (4). The COVID-19 outbreak has had a dramatic impact on China's national economy and urban social development (5). However, addressing urban public health issues requires an active response from urban spatial strategies. Therefore, the relationship between urban public health and street space is once again a hot topic of discussion.

In the urban spatial structure, the street is the most important public space in the city, which spreads like a human vascular vein (6). The management and use of urban street space has changed significantly during this epidemic. Cities around the world are constantly focusing on how to use street space wisely so that it has a positive impact on the health of their residents, carrying out street space renewal planning (organizing the Street, widening sidewalks and bike lanes, etc.) (7), strengthening street management (shared streets, safe streets, slow streets, open streets, and stay healthy streets, etc.) (8), upgrading facilities (Seattle street sink) (9), and other management measures to cope with the ongoing impact of COVID-19. However, after a long cycle of phased closures, strict travel restrictions and health protocols, the city has finally opened up and has entered a new normal. Under the new normal, how to maintain residents' health more scientifically and effectively in urban street space is still a topic that needs continuous research. At this point, a comparison of research on the relationship between street space and residents' health before and after the COVID-19 pandemic is particularly important. “This was a great opportunity to rethink how we use streets? Can we have spaces that are actually great opportunities for people to gather and gather safely? (7)”. In existing research, Sharifi (10) mapped key themes and trends in research on cities and pandemics through a bibliometric analysis, concluding research focused on six broad themes: air quality, meteorological factors, built environment factors, transportation, socio-economic disparities, and smart cities. Meanwhile, Pan and Huang (11) analyzes the measures and practices adopted by different cities in street space management during the epidemic and summarizes the impact of the post-epidemic period on urban street space planning and development. However, the present research covers a wide range of topics, is less targeted, and lacks a comparison of studies on the relationship between street space and residents' health before and after the outbreak. Therefore, an overview and trend analysis of the study on the relationship between urban streets and residents' health before and after the epidemic has positive implications for Chinese cities and even cities in other countries today.

There is no doubt that the relationship between urban streets and residents' health in China will emerge in the research of different professional disciplines. The street, as an administrative and statistical unit, can be used in the research of health management, disease investigation, and analysis, especially during the epidemic period; it will be considered as the “first line of defense for epidemic prevention and control” (12). At the same time, it will also be employed as a geospatial unit for research on streetscape, street interface, and spatial accessibility. The research results of these different disciplines objectively record the research development on the relationship between urban streets and residents' health and present them in the form of knowledge units or knowledge groups. However, if only reviewing and analyzing related literature on the relationship between urban streets and residents' health under the perspective of a single subject, it would easily ignore the implicit information such as the interaction, intersection, and evolution of various knowledge groups in the study. In general, researchers will find new research content through the relationship between these knowledge groups, to better provide a reference for frontier exploration and hot spot analysis for subsequent research. On the other hand, before and after the outbreak can be a reasonable research timeline. For the research on the relationship between urban streets and residents' health in China, it is necessary to compare the evolution of the same research topic in different periods under different disciplinary backgrounds, as well as how these themes are related meaningfully.

In this context, this study aims to review research on the relationship between urban streets and residents' health in China from 1999–2019 to 2020–2022. Specific objectives were to identify key topic areas, discuss how they have changed over time, and generate references that highlight influential literature. Through comparison, this study also emphasizes lessons to be learned from the epidemic and makes recommendations for areas that are relatively underdeveloped and require further research. This study provides a detailed systematic review of specific issues, emphasizing the relevance of various topics. The research findings can be used to understand better the current knowledge structure related to the subject, provide critical resources for reference and obtain more information for interested researchers and decision-makers, and then identify potential research differences.

## 2. Method and data

The bibliometric analysis summarizes large quantities of bibliometric data to present the state of the intellectual structure and emerging trends of a research topic or field. The analytical process is a combination of quantitative and qualitative assessment and interpretation (13–15). This study is based on the literature data regarding the relationship between urban streets and residents' health in China for 23 years before and after the epidemic. In order to guarantee the scientific accuracy of the analysis process and results, the analysis methods used need to be systematic (16). This study was analyzed by Cite Space (6.1.R3), VOSviewer (1.6.11), and Co-occurrence (COOC 13.4)^1^ (17, 18), and compared and revealed research hotspots and thematic evolution of the relationship between urban streets and residents' health in China before and after the epidemic by using keyword domain co-occurrence network, thematic evolution path analysis, and dissimilarity matrix. It then provides researchers with research frontiers, hotspots, and preliminary study contents in this field.

To make the analyzed data more comprehensive and reliable, the China National Knowledge Infrastructure (CNKI) databases was used as the data retrieval source. The database is listed as the main journal articles access database in Oxford LibGuides and Harvard Library's Chinese Studies, and CNKI is also the largest digital library in China, which includes most Chinese academic publications (19–21). The search in this database was conducted by setting the search criteria to the topic “street and resident health” or “street space and resident health,” and the periods were limited to 1999–2019 and 2020–2022, respectively. In the 1999–2019 time period, 3,304 journal articles were retrieved, and the data were cleaned (deletion of missing fields, merging of synonyms, deletion of non-sense words, etc.); the remaining 688 articles, 754 journal articles were retrieved from 2020 to 2022, and invalid articles were eliminated, leaving 210 articles. In the analysis process, the study mainly selected documents included in CSSCI (Chinese Social Sciences Citation Index) and the Chinese Core Journal Criterion of PKU database, and highly cited documents, to guarantee the reliability and validity of the analysis results.

In this study, the Dissimilarity Matrix was constructed as follows (22). Firstly, the co-word matrix was converted into a correlation matrix by the Ochiai coefficient to eliminate the influence of the co-word matrix due to the significant difference in frequency. Among them, $Ochiai=\frac{n\left(AI\;B\right)}{\sqrt{n\left(A\right)\times n\left(B\right)}}$, the individual data in the correlation matrix can then be subtracted by 1 to form a table of dissimilarity matrices, $Dissimilarity\;Matrix=1-\frac{n\left(AI\;B\right)}{\sqrt{n\left(A\right)\times n\left(B\right)}}$.

## 3. Results and discussions

According to the above, the paper sorts out the literature focus and research fields in these two periods, then discuss the research trend, the main topic areas of research, and the results of the analysis of the evolution path of the research topic.

### 3.1. Research trends

**Figure 1:**
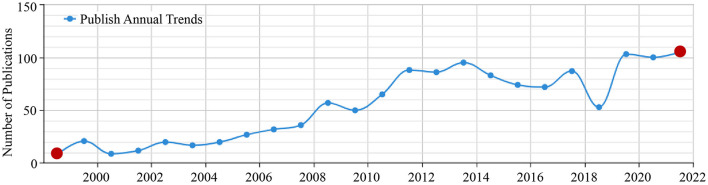
Temporal distribution of the number of publications on the relationship between urban streets and residents' health in China from 1999 to 2022.

As seen in Figure 1, the overall research literature on the relationship between urban streets and residents' health in China during 1999–2019 showed an increasing trend year by year. The research in this period can be divided into 3 phases based on the number of publications. 1999–2007 belonged to the slow development period, with a slight increase in the number of literature each year. Starting from 2008, research on the topic entered a period of development, maintaining a steady growth trend from 2012 to 2015, reaching its highest value around 2014 and a low value during 2018–2019, followed by an incremental increase. The overall trend in the number of publications shows a general trend of increasing interest in the topic from year to year among researchers.

The study of the relationship between urban streets and residents' health in China, as a cross-cutting research theme, mainly involves the following disciplines during 1999–2019: public health and preventive medicine, clinical medicine, society, politics, urban and rural planning and municipalities, urban economy, and environment (in order of the percentage of literature by discipline); during 2020–2022, it mainly involves public health and preventive medicine, the urban and rural planning and municipality, urban economy, society, politics, etc. There is a clear shift on the proportion of literature by various disciplines and particularly with a significant increase in urban and rural planning and municipalities, and urban economy between 2020 and 2022.

### 3.2. Main thematic focus areas of research

#### 3.2.1. 1999–2019

**Figure 2:**
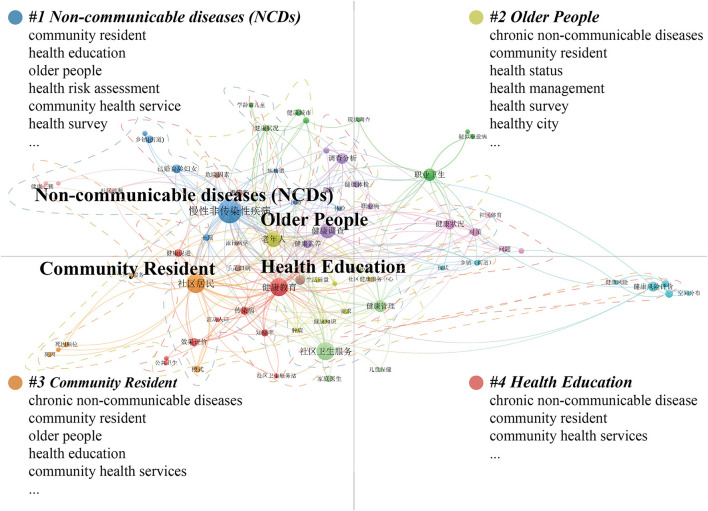
Major thematic focus areas for 1999–2019.

Valid keywords were extracted from 688 articles, and a total of 1,575 keywords were obtained. Figure 2 shows the co-occurrence spectrum of 72 high-frequency keywords that appeared four times or more, and the higher the frequency of the keywords, the more important the keywords are in the research field (their nodes and centrality are larger). This map constitutes the basic direction of the research on the relationship between urban streets and residents' health in China from 1999 to 2019, closely dovetailing with the content of the research field itself and the essential elements that constitute it. Based on two indicators, occurrences, and total link strength, four main topic areas were identified: non-communicable diseases (NCDs), community residents, health education, and older people.

#### 3.2.2. 2020–2022

**Figure 3:**
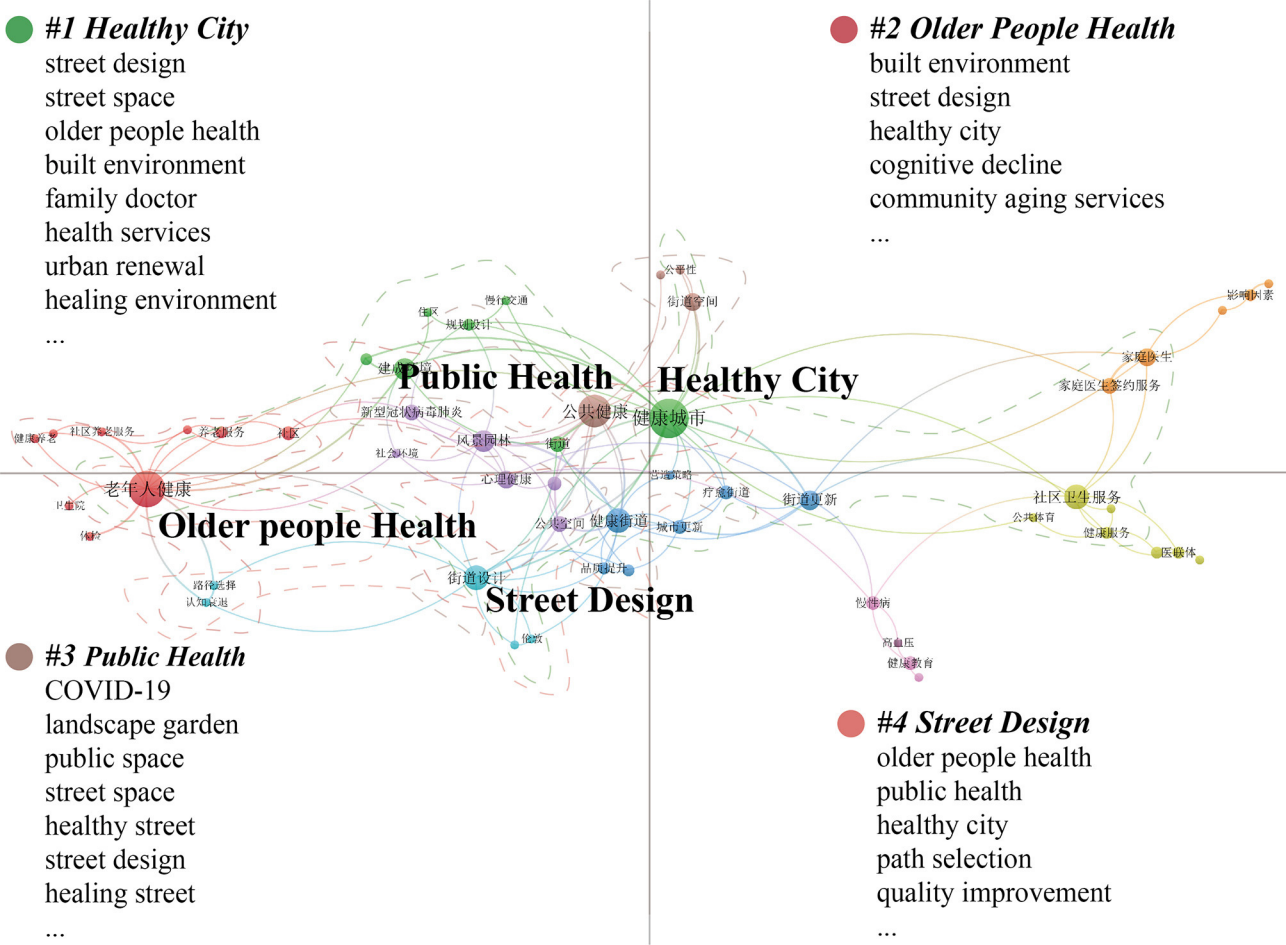
Major thematic focus areas for 2020–2022.

Valid keywords were extracted from 210 papers, and a total of 677 keywords were obtained. Figure 3 shows the co-occurrence spectrum of 63 high-frequency keywords with two or more occurrences. This map constitutes the basic direction of research on the relationship between urban streets and residents' health in China from 2020 to 2022, and it also clearly reflects the four main topic areas: healthy city, older people health, public health, and street design in this time period.

### 3.3. Analysis of the evolutionary path of research themes

**Figure 4:**
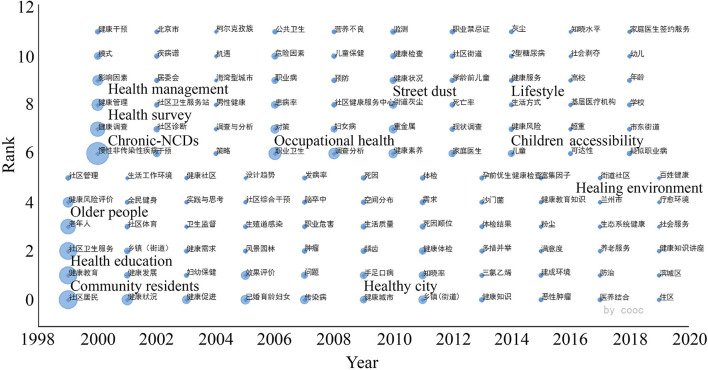
Cumulative time zone diagram of the evolution of research themes 1999–2019.

**Figure 5:**
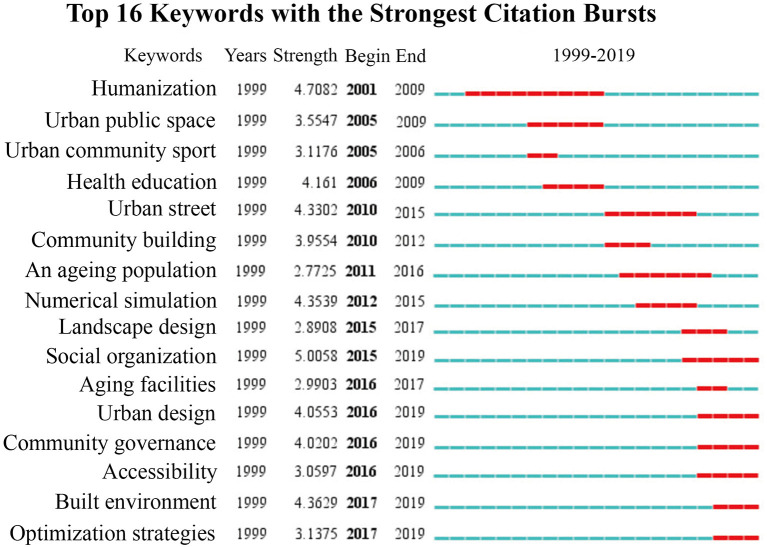
List of emergent words for research themes 1999–2019.

In this study, a time-zone diagram of the theme evolution was analyzed by COOC, and Burst Detection was performed by CiteSpace, which is based on the temporal dimension to determine (grasp) the research evolution paths, evolution characteristics, and frontier research trends of the research theme over different periods. Figure 4 shows the thematic evolution trend of the research topic over 20 years. Figure 5 lists the first 16 emergent words of this research theme, and the intensity of the emergent words and their starting and ending times can be seen in the table. The changes in the mutated words indicate that the theme of this study has gradually shifted from perspectives such as humanized design and physical activity promotion to shared decision-making approaches such as social organization participation and governance; the change from urban public space to urban design, with more attention to the combination of various relationships and the cross-integration of subsystems affecting the relationship between urban streets and residents' health in China, and the shift to system design. In this study, Figures 4, 5 are analyzed together to obtain three stages of the evolution of this research theme.

During 1999–2009, the emergent terms with high salience and centrality were humanization, urban public space, and urban community sports. The emergent terms in this period indicate that research on the relationship between urban streets and residents' health in China has focused on the humanization of spaces, the development of community sports, and the education and management of residents' health. Residents' daily hygienic behavior is the critical normative content of health education through the production and distribution of promotional materials (bulletin boards, leaflets), explaining to residents about the prevention and treatment of chronic diseases, and guiding them to look at hygiene bulletin boards, hygiene wall posters, etc., and regularly carrying out thematic hygiene knowledge competitions and health consultation activities (23). Along with the accelerated urbanization process, urban public space has been occupied by commercial development, and the large-scale functionalization of the area has caused the city to lose its original texture and vitality. The spatial measure is based on people's “scale of daily life” (behavioral scale, psychological scale) (24). It can be seen that the background of this period was the gradual acceleration of urbanization. It was an important issue at that time to overcome the rough and loose development mode and to make the socio-economic, demographic, and urban environment develop harmoniously and maintain a healthy operation.

During 2010–2015, urban streets, community construction, population aging, and landscape design were the emergent terms with high emergence and centrality. Based on the SD method, Gou and Wang (25) evaluated the spatial vitality of streets in terms of human perceptions. They concluded that effective ways to make streets more vital: improving accessibility, the comfort of the street environment, and meeting the diverse needs of residents. Li et al. (26) argued that at that time, Chinese cities built and renovated large areas of neighborhoods mainly to enhance the city's image and promote economic growth, which made the neighborhoods lose their significance as places of living space. Gallagher (27) argues that Chinese urban streets are oriented to prioritize motor vehicles, neglecting streets as an important part of urban public space, and emphasizes that the whole process should be finely guided, and responsibilities must be clarified; relevant design code standards should be developed; and the concept of streets as urban public space has to be changed. The research theme has entered a stage of in-depth development, at which time more targeted research content has been produced, and corresponding responses have been made to the overdevelopment triggered by the one-sided pursuit of commercial interests that emerged in the rapid urbanization process, indicating to a certain extent that the research theme is in an active stage.

During 2016–2019, only the words of social organization, elderly service facilities, urban design, community governance, accessibility, built environment, and optimization strategy emerged. Luo and Fu (28), based on the stratified needs, the proposed configuration facilities of community parks are divided into standard, optional, and tentative configuration items. However, the study was conducted in Shanghai, a first-tier city in China, and the findings have limited implications for other levels of cities and villages given the varying degrees of aging, socioeconomic levels, and infrastructure in different levels of cities. Sun and Yin (29) argue that the occupants of demolition and resettlement houses are not free to choose their place of residence, the effect of residence self-selection was stripped out and the effect of the built-up environment (population density, parking lot density, street intersection density, and subway station density) on residents' physical and mental health was explored by multiple regression in Chinese cities. The age group (post-80s) and the lack of health dimensions (social health) are the limitations of this study. Xu et al. (30) demonstrated through an experimental approach of virtual streets that architectural interfaces affect the human experience in the street environment more than the effect of green view rate. However, virtual immersion does not fully reproduce the experience of the real environment, and many important environmental factors are shielded. Chen et al. (31) studied the factors of streetscape quality (greenery, architecture, pavement, public facilities, color, soundscape, and culture) that affect human psychological emotions is based on a subjective assessment method, but more objective physiological indicators (heart rate, blood pressure, and skin temperature) are needed to study human emotional perceptions of different streetscape environments. Tang et al. (32) assessed the overall spatial quality of streets through streetscape images as a basis for reflecting pedestrians' intention to stay. Some other authors have studied the association between street space microclimate, public green space, and residents' health. Shao and Liu (33) reviewed five factors (street airflow, temperature, shading, pollutants, and humidity) that affected street climate and summarized the associations between these influencing factors. Yu et al. (34) shared fitness data based on a cell phone app study, and the results showed that street space continuity helps to promote fitness activities, and large parks and colleges are attractive to residents' fitness activities.

The context of the study theme at this time is the emergence of new types of research evidence as cities enter a phase of stock renewal and a new phase of centralized data generation for all types of urban data. Big data and open data constitute the new urban data environment, making the research results more scientific and overcoming the limitations of static, scale, and granularity trade-offs in traditional methods. However, numerous researchers are at the stage of analyzing the data and exploring the relationship between variables, and the evaluation of how to make the research results serve in the actual street space update and the effects after use is relatively weak. On the other hand, the equity of the study also needs to be concerned, as most of the studies focus on first-tier cities with good economic environments and infrastructure, and more levels of cities are needed as research subjects.

**Figure 6:**
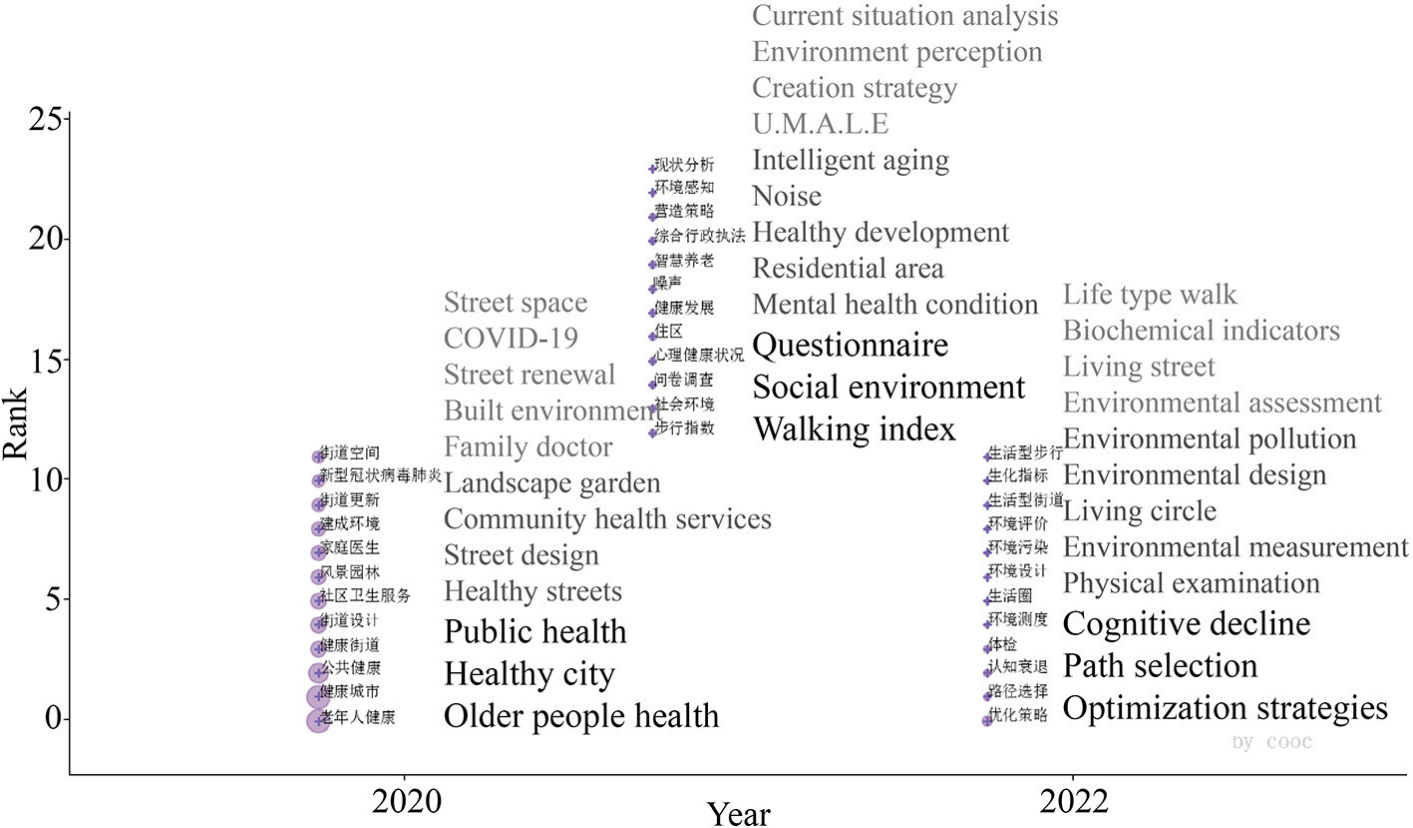
Cumulative time zone diagram of the evolution of research themes 2020–2022.

**Figure 7:**
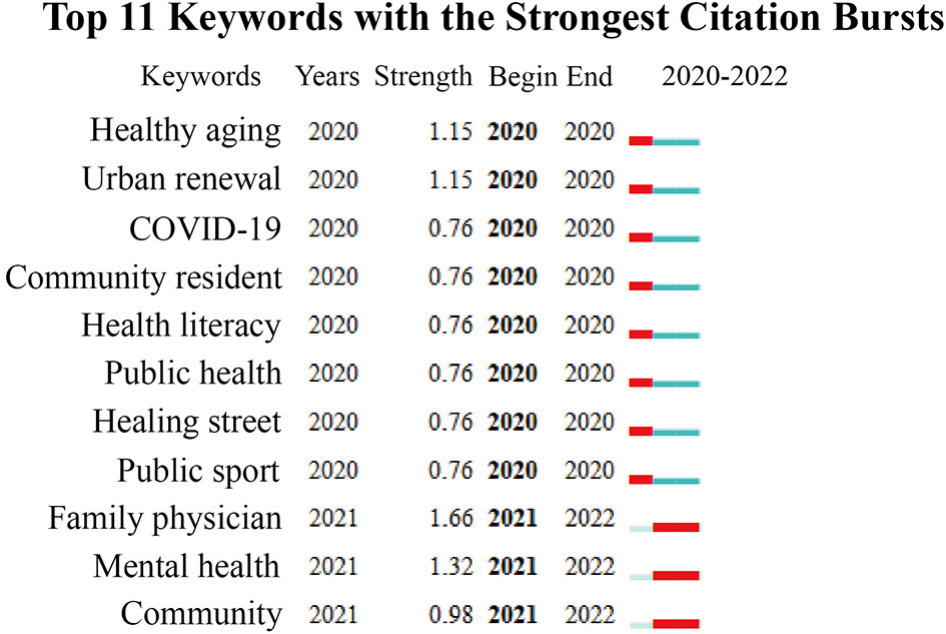
List of emergent words for research themes 2020–2022.

The thematic evolution trends of this research area in the period of 2020–2022 are shown in Figure 6, and the 11 mutation terms for that period are listed in Figure 7. A joint analysis of Figures 6, 7 yielded that healthy aging, public health, and COVID-19 were prominent in 2020; mental health and family physicians were prominent in 2021. Affected by COVID-19 during this period, researchers accelerated the search for evidence to improve population health and enhance public health.

### 3.4. Constructing dissimilarity matrix

#### 3.4.1. 1999–2019

**Figure 8:**
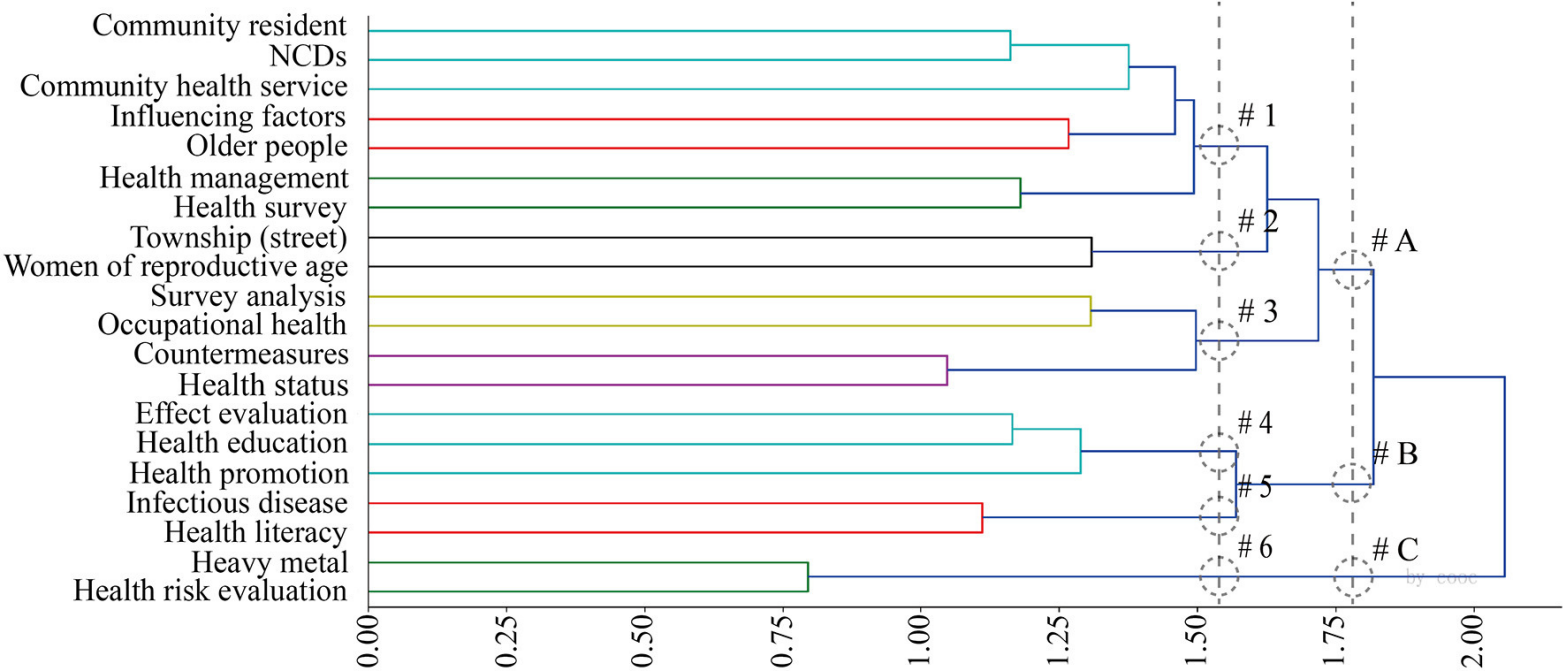
Hierarchical cluster analysis dendrogram 1999–2019.

**Figure 9:**
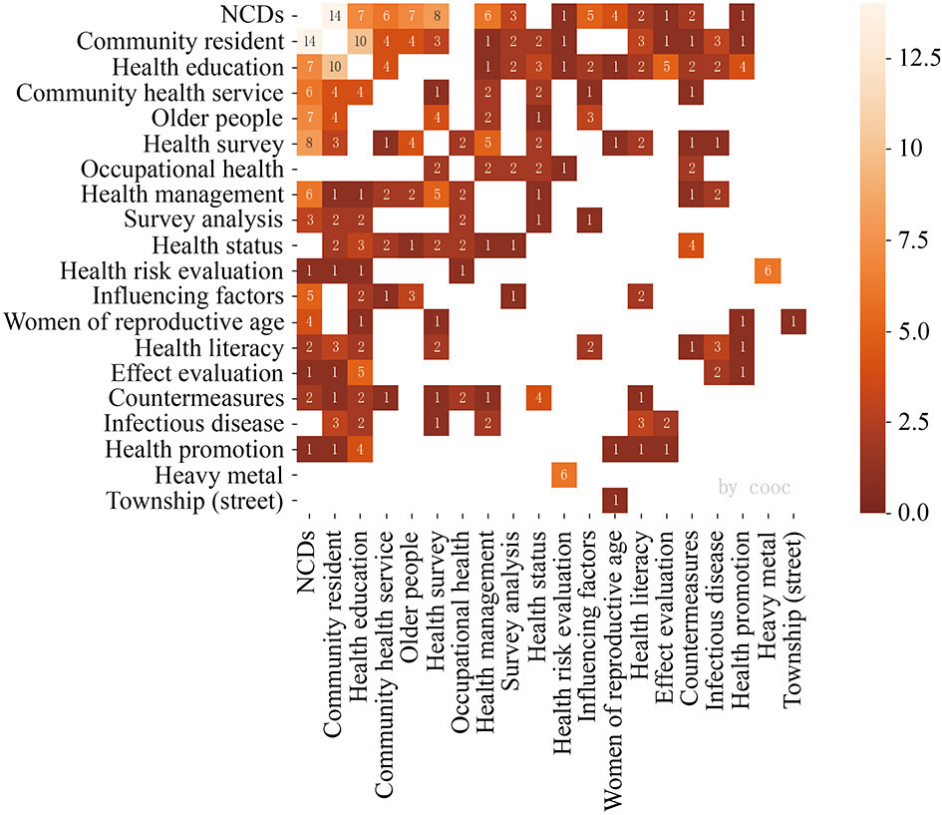
Confusion matrix, 1999–2019.

The clustering tree diagram (Figure 8) and confusion matrix diagram (Figure 9) were obtained by COOC calculation, and these two diagrams were combined and analyzed, and three major categories and six subcategories were integrated by reading related literature under different topics. The first subcategory ranged from community residents to health surveys, and the studies were mostly management and service-oriented, involving the general population, the elderly, and also emphasizing the attention to other disadvantaged groups such as lost families. Huang (35) believed that the government should pay more attention to and help the families who lost their independence when purchasing public services. Han and Sun (36) found that public service purchases are not necessarily conducive to the effectiveness of public service acquisitions because of the pursuit of “pure market” and “pure social” practices; government purchases only complete the socialization of public service “production”, but not the socialization of public service “production”. The study also responds to the phenomenon of “reverse contract outsourcing”, which provides a realistic reference and a method for “Chinese style public service purchasing” during the transition period. The study replies to the phenomenon of “reverse contract outsourcing” and provides a realistic reference and a theoretical interpretation of “Chinese public service purchasing” in the transition period. Chen et al. (37) study the equity of spatial distribution of health arising from different types of spatial distribution for people of different age structures on an exposure perspective. On the other hand, Chen et al. (38) proposed the construction of a four-in-one diversified elderly care pattern of government, family, society, and community from the deep integration of the Internet, networked services, and intelligent elderly care.

The second subcategory contains only township (street) and women of reproductive age. The third subcategory is from survey analysis to health status. In these two categories, streets appear as administrative services and statistical units. The research focuses on the reproductive health and health knowledge of married women of childbearing age (39, 40), as well as the investigation and analysis of the occupational health status of various types of practitioners. The study emphasizes that basic occupational health services in urban grassroots public health units play an important role in the prevention and intervention of occupational disease hazards. The fourth and sixth subcategories range from infectious disease to health risk evaluation, in which streets are often used as statistical units and geospatial units. Xue et al. (41) argued that an early warning system using advancing spatial-temporal rearrangement scan statistics combined with geographic information software could be effective in making early warnings of infectious disease outbreaks. On the other hand, Hu et al. (42) introduced a procedure for extracting heavy metal forms in atmospheric dust. Zhang et al. (43) analyzed and evaluated the spatial distribution characteristics of lead content levels in street dust in downtown and suburban town centers of Shanghai.

Integrating and analyzing the results of these three categories mentioned above, it was obtained that the literature is dominated by studies focusing on community residents, health surveys, and health education. In other words, research questions related to urban public health are relatively under-explored. An important issue that can be noted from this analysis is the diversity of groups targeted for study in health survey research (orphaned families, women, grassroots practitioners, etc.), which brings insight into public health research from an urban planning and urban design perspective (there is a homogeneity of research groups). The change in the content of health education requires a diversification of the means of communication and the timeliness of acceptance by residents throughout the process.

#### 3.4.2. 2020–2022

**Figure 10:**
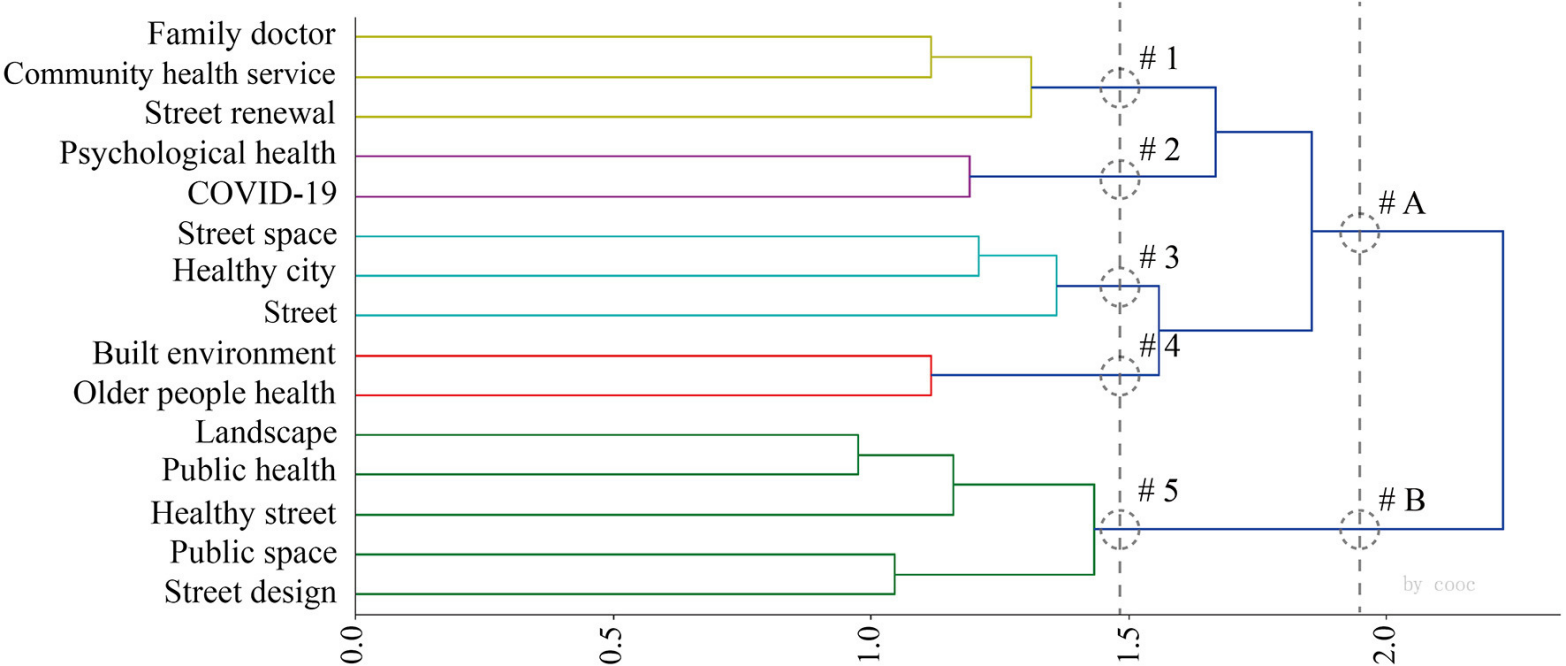
Hierarchical cluster analysis dendrogram 2020–2022.

**Figure 11:**
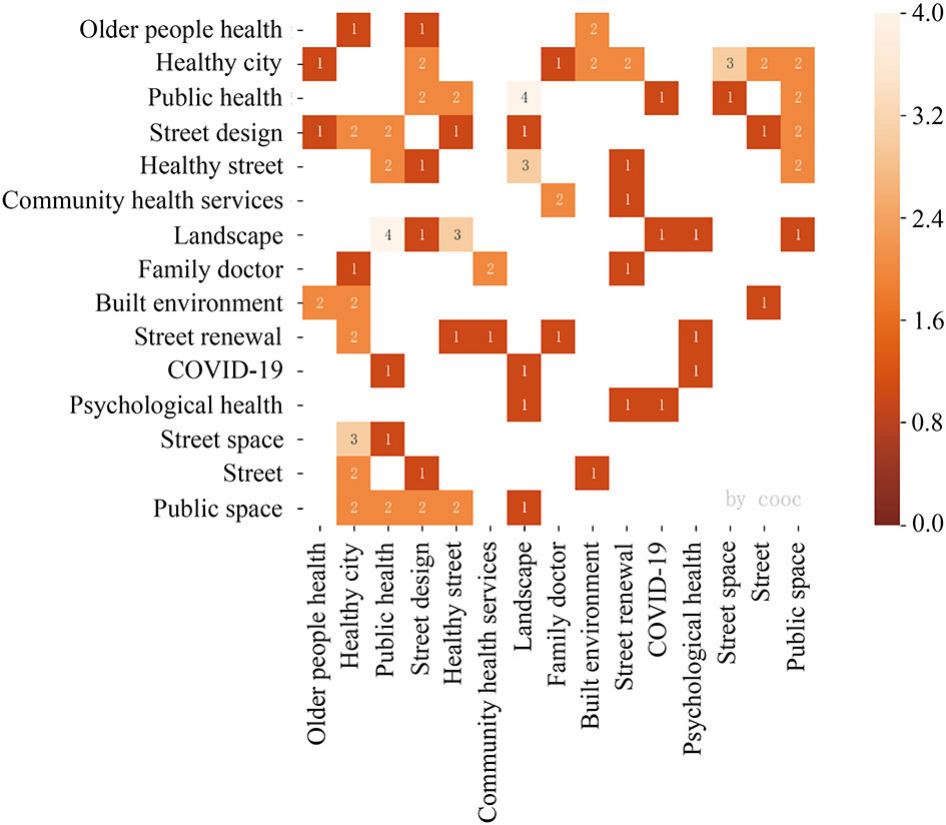
Confusion matrix, 2020–2022.

The clustering tree diagram (Figure 10) and the confusion matrix diagram (Figure 11) were combined and analyzed, and two major categories and five subcategories were integrated by reading related literature under different topics. The first subcategory ranges from family doctor to street renewal, and Huang (44) argues that residents have differentiated needs for family doctor contracted services, and should provide personalized and diversified services, establish stable contracted service relationships, attract residents to sink to the clinic, and guide the orderly and efficient hierarchical diagnosis and treatment pattern. Guo and Dai (45) found that the structural dilemma of urban grassroots community governance lies in the government's inertia in “seeking stability”, the limited development of social organizations, and the lack of supportive policies.

The second subcategory contained only psychological health and COVID-19, and He et al. (46) concluded that anxiety, depression, and their co-morbidities among adult residents during COVID-19 were caused by different risk streets, years of work, alcohol consumption, and underlying illnesses. However, the study did not mention the impact of residents' economic income and how interventions such as psychological counseling and mental health education were developed and implemented. Xu et al. (47) concluded that the epidemic significantly affected the time allocation of various types of activities of residents, and these effects include authority constraints, combination constraints, and ability constraints on residents' spatiotemporal behaviors which are differed significantly at different stages of the epidemic development, and analyzed the changes in residents' behaviors from the norm to the extraordinary state and from the emergency state to the norm. However, the specific effects and mechanisms of COVID-19 on the daily activities of individuals and households, as well as the study of the differences among different populations and households, still need to be followed by a large number of case studies. Peng et al. (48) studied the geographical distribution of confirmed COVID-19 cases in combination with urban traffic data and concluded that the effect of road network density on epidemic transmission was not significant, and there was a significant positive correlation between the distribution of rail transit stations and epidemic transmission, i.e., rail transit routes may become “high- speed corridors” for epidemic transmission in cities. However, there is a negative correlation between road accessibility and epidemic transmission. Yan et al. (49) argue that group prevention, data-driven, technology-assisted prevention, and service-led prevention are important factors supporting the effective operation of agile prevention and controlling mechanisms at the grassroots level, but a series of practical dilemmas still exist, such as group prevention is not widespread and data-driven is not comprehensive. In this category, the researchers analyzed the impact of COVID-19 on residents' health and epidemic transmission prevention and control in terms of residents' own situation, government policy situation and urban traffic operation. At this point, other researchers are reminded that they should not merely use big data to assist the intelligent management of cities after the outbreak, where it focuses not only on monitoring current situation but also emphasizing dynamic early warning and employing various types of data for analysis to guide the formulation of urban epidemic prevention and control strategies.

In the third and fourth subcategories, from street space to older people health, Yu et al. (50) proposed the concept of healthy streets and the mechanism of action from the dimensions of physical health, psychological health, and social adaptation, and identified four health impact paths of physical activity, environmental comfort, street safety, and social interaction. Zhang et al. (51) suggested that permeability characteristics such as land use density, functional mix, road network density, and permeability along the street were positively correlated with theft density as a whole. In communities with stable social structures, street eyes can play a natural monitoring role, and the explanatory power of the street eye theory is stronger, but such communities are still a minority in real life, and blind adoption of the New Urbanist design approach may exacerbate security risks. Chen et al. (52) used multi-source data such as streetscape images and other technical methods for instance off-site built environment audit to explore the phenomenon of spatial disorder in Hefei city and the relationship between different types of streets and the degree of spatial disorder, which provides an important basis for refined management of the city in the future. Ta et al. (53) measured the economic, social, and cultural dimensions of urban vitality by using data from public reviews, cab arrival and cultural facilities POI, analyzed the spatial structure characteristics of urban vitality at the neighborhood and street levels, and established an econometric model to analyze the relationship between urban built environment and urban vitality. Si et al. (54) used streetscape data, road network data, POI data, and mobile Internet location service data to develop a weekend time-of-day model on exploring the spatial and temporal distribution characteristics of commercial and living street vitality and the influence of the built environment.

On the other hand, Yu et al. (55) discussed the impact paths of urban streets on public health. They sorted out five types of spatial elements and measures of street traffic, interface, space, greenery, and facilities under different impact paths. Han et al. (56) found that in terms of street livability, street amenity density promotes leisure and social activity trips; according to street aesthetics, street greenery positively moderates the ability of older adults to perform daily chores, and interface continuity helps increase the frequency of leisure and social activities. Wang and Yang (57) suggested that a high-quality green space environment, mixing with residential land, proximity to amenity streets, and the layout of diversified outlets in the surrounding area can positively regulate the frequency of green space use for older people. Yuan and Chen (58) concluded that the street environment influences the walking behavior of cognitively declining older adults in a different way than healthy older people, with the environmental variables in descending order of influence: street scale, street interface, road network configuration, street function, and street furniture.

In the fifth subcategory, from landscape to street design, Li and Yang (59) propose a framework of hot topics for landscape gardening focusing on public health in the post-epidemic era, including seven specific directions in three aspects: in planning and design, focus on optimizing the park green space system based on the community living circle, building diversified neighborhood green places emphasizing usability, and strengthening green space in the park as a reserved space for epidemic prevention and emergency services. For management, it will focus on the operation of health improvement projects and events in parks and green spaces and build a refined management system for parks and green spaces with an emphasis on public health. For the security mechanisms, benefit assessments of public health improvement need to be conducted, the sources of funding and service providers has to be expanded, and multiparty cooperation in the new public health era will be deeply and proactively participated in. Li and Xu (60) argue that healing environments have expanded from traditional healing gardens and landscapes to a variety of environmental areas such as healing streets, healing architecture, and virtual healing. They also point out that healing environments should be integrated into the public health system to gain new momentum as part of the healthy city movement. Tan (61) analyzes current problems in public health from the perspective of risk society, highlighting the lack of risk awareness in urban street communities and the insufficient attention to public health, the imperfect legal and regulatory system of public health in communities, and unfavorable education and publicity of community health and the rule of law; the weakness of community crisis prevention and public health forces; and insufficient effectiveness of multiple subjects in public health, etc.

During this period, research related to COVID-19, healthy city, and public health topics were more prominent. Among them, the healthy street is a new model for street development and a new direction for street design in the context of healthy city and stock planning, and it provides physical, psychological, and social dimensions of health services for residents. During a pandemic, the combination of administrative and geospatial attributes of streets is more evident, and the epidemic policies at different stages influence when and where residents go out and have a subtle impact on their daily life behaviors, for example, online food delivery services (56), communities turn to self-help and mutual aid as a way to address emergency needs, etc.

On the other hand, for this topic, searches in two databases, web of science and ScienceDirect, found that travel restrictions imposed during the pandemic increased the risk of alienation and associated stress and anxiety issues. At a time when most residents want to maintain their physical and mental health by accessing parks and street green spaces (62–64), it is particularly important to keep effective access to parks and to preserve the equity of green space distribution (65). In addition, Barbarossa (66) believes that providing more space for active transportation (bicycling, walking) will also help prevent the overloading of the public transportation system and thus better counteract future epidemics. Bike lanes and pedestrian paths should be integrated with the city's green infrastructure network to make the environment more appealing and provide health/adaptation co-benefits (67). However, the purpose of street reallocations is not only to benefit bicycling residents but to allow residents to have more opportunities to exercise outdoors and access essential commercial services (68, 69). These reallocation decisions may discourage other mobility needs (e.g., essential workers, deliveries) (70). Accordingly, it is particularly important to maintain mobility, justice, and equity in the use of street space.

Another important issue reflected by the pandemic is the vulnerability of cities to supply chain disruptions, especially those related to the food supply, mainly due to restrictions on movement in pandemic prevention policies that block the food transportation chain from farms to markets and to residents' homes, which is undoubtedly a huge blow to low-income groups and the unemployed (71). Lal (72) and Langemeyer et al. (73) argue that home gardening, urban agriculture, and the distribution of vegetable gardens helped to mitigate the impact of the COVID-19 pandemic on food security. The comparison shows that the current literature on this topic in CNKI focuses more on the physical and mental health of the population, while the research on the social health of the population is weak and not in-depth enough, in particular, research topics are not critical and diverse. Consequently, there is a strong need to increase research topics focusing on vulnerable groups.

**Figure 12:**
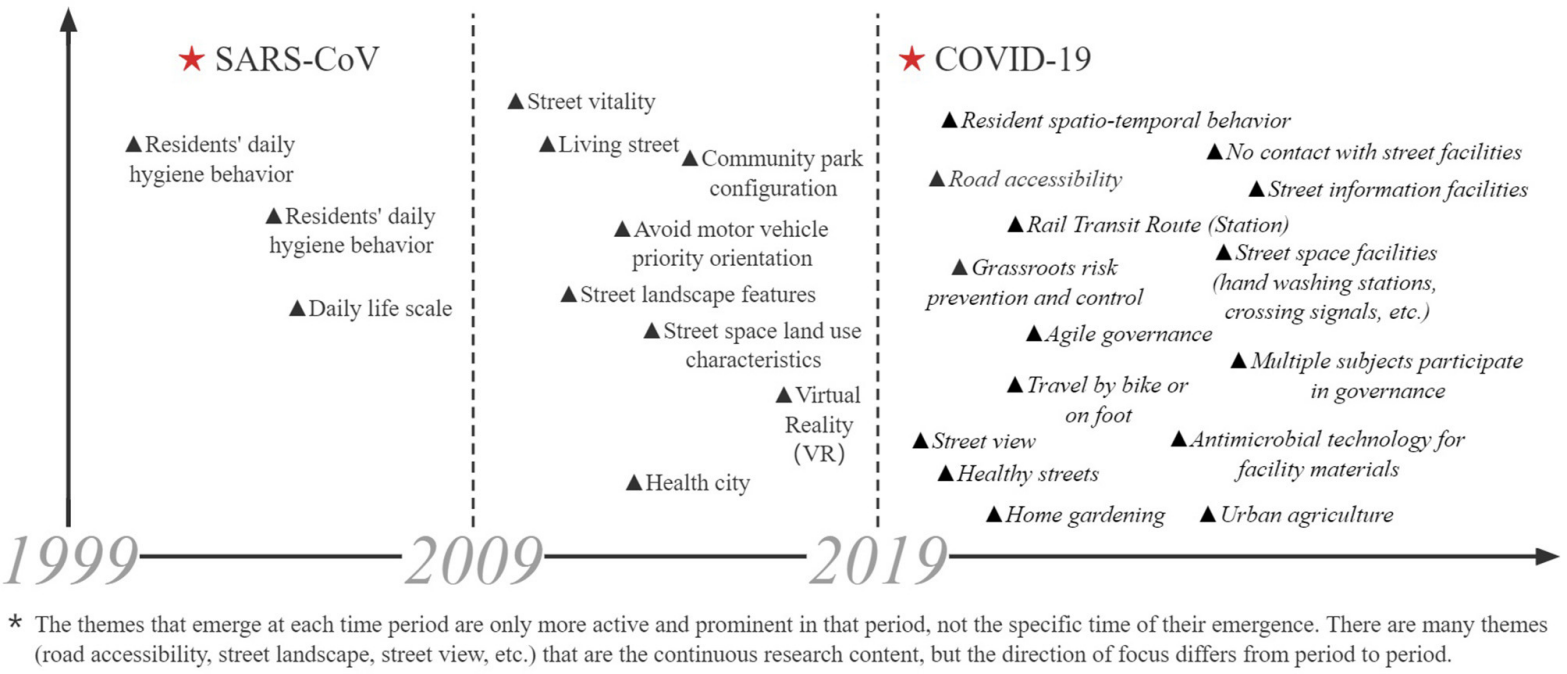
Trend of theme change by period.

From before to after COVID-19, the theme extended from the focus on the health of community residents and the sustainable development of healthy streets to re-examine the allocation of street space and refined management, focusing on women and vulnerable groups (the older people, the unemployed, the homeless, etc.), this has accumulated more experiences and rich research directions for exploring the relationship between street space and residents' health such as building more humane streets and improving spatial compatibility; adhering to the orientation of healthy street planning and design, and refining the design of street facilities; using flexible thinking to manage street space in multiple dimensions and pluralistic participation in governance (civil society groups); continuing to pay more attention to women and disadvantaged groups; applying urban multi-source data to improve the information and technology of street governance, etc. Table 1 lists the relatively mature research areas and under-explored areas of these six topics. Figure 12 shows the changing trend of this theme.

**Table 1:** The list of the relatively mature research areas and under-explored areas.

**Time**	**Theme**	Relatively mature research areas^*^	**Under-explored areas**
1999–2019	Community residents	·Older people (aging services, chronic diseases) ·Married women of childbearing age (reproductive health) ·Various types of practitioners (occupational diseases, status quo policy)	·Specific physical and mental illnesses
	Health survey	·Health literacy ·Health e-portfolio ·Chronic diseases	·Chronic disease management strategies
	Health education	·Group peer education, expert guidance ·Primary health care ·Community health service center	·Health education and promotion on administrative and operational organization ·Health education and promotion platform ·Health education information technology ·Participatory health education
2020–2022	COVID-19	·Psychological health ·Agile prevention and control mechanisms at the grassroots level ·Spatiotemporal behavior ·Traffic data	·Medium- and long-term impacts on the urban economy ·Green travel
	Healthy city	·Street view images ·Built environment ·Healing landscape	·Long-term impacts ·New urban governance ·Micro- political interactions ·Long-term structural health inequalities
	Public health	·Family doctor ·Street space elements ·Park green space ·Street community risk awareness	·Privacy and data security ·Governance subjects (enterprise, social organizations, public)

^*^Since this topic belongs to the content of interdisciplinary research, the theme “Relatively mature research areas” is not entirely fitted with a specific theme. For example, “park green space” might appear in “healthy city” and “COVID-19”.

## 4. Conclusions

The impact of COVID-19 has left cities operating in an environment with extreme uncertainty. Such a result is a challenge to the socio-economic, public health, and social stability, leaving governments with difficult trade-offs. City managers and researchers have explored different perspectives on how to control the spread of pandemics in urban spaces to safeguard sustainable urban development and health equity for residents; among them, the street is the most basic research unit. Although human society has been affected by COVID-19, the relationship between street space and residents' health should also follow the objective laws of urban, economic, and social development. A paradigm shift in governance from planning concepts and design tools to the policy of management is needed, rather than relying on a temporary and campaign approach to deal with the relationship between the two when a public health event occurs, and such shift must become a regular and continuous spatial governance effort.

The purpose of this study is to provide an overview of existing research on the relationship between street and resident health in urban China before and after the onset of COVID-19, to identify major topic areas, and to highlight under-researched areas. In an analysis of 688 papers published from 1999 to 2019, it was found that research relating to this topic can be summarized into three major themes: community residents, health surveys, and health education. And from 2020 to 2022, themes of COVID-19, healthy city, and public health were dominated, and the research relating to this topic is enriching and growing.

COVID-19 once again reveals how pandemics change the spatial structure of cities and social relations in space, reshaping space and social relations during the pandemic, which also created new spatial inequalities. Hence, executable policy of health is depending on political will and state budgeting (3). When studying the relationship between street space and residential health, it should strengthen the “Cross organizational cooperation and interaction” between local governments, social service organizations, self-organized civil networks, and health institutions. This will stimulate potential institutional capacity to respond to pandemics and maintain bottom-up resilience as well as civic resilience. On the other hand, the interdisciplinary (urban design, geography, social design, public health, political science, sociology, etc.) collaborative research and technological interventions are also worth considering, for example, the tracking of infected people's activities, the delineation of high-risk zones in cities and the study of urban streetscape data, all through basic urban data (basic traffic data, basic urban land data, etc.), internet open source data (public facilities data, urban weather data, etc.) and commercial level data (personal mobile device data, personal travel data, multidimensional personal profile, etc.) can be applied to achieve this goal. Therefore, how to achieve interdisciplinary cooperation and effective access to multi-source data will be the ongoing topic of research development in the future. In addition, the construction of assisting path on the accuracy of choosing study direction for researchers is also a considerable and potentially extended research field.

Overall, the pandemic has had a subtle impact on the relationship between streets and the health of China's residents. Now that 3 years have passed since the pandemic, it is the appropriate time to take stock of the lessons learned in order to improve the ability of cities to cope with the pandemic and to maintain sustainable and healthy urban development. First, because the streets have different orientations in the research of different disciplines, but they are interrelated, so the theoretical framework, research methods and research paradigms of the research are diverse. Second, at different times, the integration of urban streets and residents' health is different, and the correlation between the resources of each element within this complex system has not been sufficiently studied. Third, the shift of the study from relevance exploration to the causality of the research content, makes the findings more convincing. Fourth, there have been researchers who have conducted quantitative research through experimental methods, using technical equipment and virtual experiments, but the complex diversity of the city itself makes the research results remain some uncertainties.

The research on the relationship between urban streets and residents' health is influenced by multiple factors such as urban economy, politics, society, science and technology, public health, etc. The research should be based on the actual situation of urban development, with more links to administrative management, public health, pathology, and other disciplines to strengthen the relationship, distinguish the problem under study and discern its characteristics. In the process of problem analysis, it is important to avoid the reduction of interdisciplinary cross-research opportunities due to the lack of knowledge on related research contents in different disciplines, which is one of the reasons why it is difficult to implement research and obtain appropriate results. Moreover, some research results, because it takes a certain period of time to execute on the ground, are less effective, and it is an issue worthy of attention on how to promote the implementation of research results. Therefore, when conducting research on the relationship between urban streets and residents' health, it is necessary to integrate the interrelationship between research elements, go beyond the traditional research from the perspective of the research object itself, and break the research paradigm of “current situation - problem - countermeasure”.

However, this study has its limitations. Evidently, bibliometric analysis is a valid method to provide an overview of the research field, identify major subject areas, and explore the evolution of themes and concepts. Yet, this study only provides a systematic review of the literature in China, and relevant research literature from other countries should be examined to better understand the impact of pandemics in providing more specific policy recommendations. In addition, most of the articles that this study analyzed are peer-reviewed. Evidence reported in the gray literature should also be considered in future studies to ensure better coverage of real-world impacts and policy-focused reporting, which will improve the coverage and breadth of study results where limitations can be reduced and reflect the needs of the real world.

## Data availability statement

The datasets presented in this study can be found in online repositories. The names of the repository/repositories and accession number(s) can be found in the article/supplementary material.

## Author contributions

LM and K-HW: conceptualization, methodology, and writing—review and editing. LM: writing—original draft, software, formal analysis, data curation, and visualization. Both authors have read and agreed to the published version of the manuscript.

## Footnotes

^1^The analysis software was developed by Xueshudiandi Team from China.
